# Racial Equity Impact Assessments as Tools for Advancing Population Health and Equity in Local Policy

**DOI:** 10.1111/1468-0009.70094

**Published:** 2026-05-24

**Authors:** KELLEE WHITE WHILBY, MAKEDA WALELO, HERON BONDOC

**Affiliations:** ^1^ School of Public Health University of Maryland College Park; ^2^ University of Maryland College Park

## Abstract

**Policy Points**

- Racial equity impact assessments (REIAs) provide policymakers with systematic evidence on how proposed legislation may affect racial equity and subsequently shape structural determinants of health.
- Across 409 bill‐level equity impact ratings derived from 296 final REIAs from 2021 to 2024, nearly half of REIAs (46%) indicated that proposed legislation would likely advance racial equity, 9% signaled potential adverse effects, 10% were neutral, and one‐third were inconclusive or negligible.
- REIAs provide an opportunity to assess potential equity impacts prior to enactment, informing policy design by helping policymakers anticipate harms and consider revisions that may support more equitable policy processes.
- The effectiveness of REIAs depends on a supportive political context and accountability structures, as adverse findings alone do not guarantee legislative action to mitigate inequities.

**Context:**

Racial equity impact assessments (REIAs) are used by local governments to integrate equity considerations into policymaking and decision‐making processes by evaluating potential impacts of proposed legislation before enactment. Despite their growing adoption, limited empirical evidence exists on how REIA findings are characterized or how equity‐focused evidence is taken up within legislative processes.

**Methods:**

A descriptive analysis of all racial equity impact assessments (REIAs) conducted in the District of Columbia between 2021 and 2024 (*n* = 296 REIAs; 409 bill‐level equity impact ratings). REIA ratings were categorized as positive, adverse, neutral, or inconclusive/negligible and analyzed across eight policy domains (e.g., health and human services, criminal justice and public safety, budget and fiscal policy). Legislative outcomes for bills with adverse ratings were classified as enacted with modification, enacted without modification, or not enacted.

**Findings:**

Nearly half (46.4%) of REIA ratings indicated positive equity impacts, while 9.0% identified potential adverse effects; 34% were inconclusive/negligible. Equity impacts varied substantially by policy domain, with positive findings concentrated in health and human services and economic policy and adverse findings more common in criminal justice, environmental, and infrastructure domains. Among bills with adverse ratings, 62.2% were enacted with modifications, 24.3% without modification, and 13.5% were not enacted.

**Conclusions:**

REIAs provide a mechanism for identifying potential policy equity impacts prior to enactment, but adverse findings alone do not guarantee legislative action. Strengthening institutional accountability structures and the integration of equity evidence into decision‐making processes is critical to ensuring that identified harms lead to meaningful policy change and to maximizing the effectiveness of REIAs as a tool to advance population health equity.

Structural racism shapes population health through policies, institutional practices, and decision‐making processes that determine access to resources and opportunities.^1^, ^2^ In response, federal, state, and local governments have increasingly adopted racial equity tools intended to integrate equity considerations into policy development and decision‐making processes to address and mitigate longstanding inequities.^3^, ^4^, ^5^, ^6^ Among these tools, racial equity impact assessments (REIAs) have emerged as a prominent strategy for anticipating whether proposed legislation, budget decisions, or programs are likely to exacerbate or reduce racial inequities.^5^ Similar in form to fiscal or environmental impact statements, REIAs provide law makers with systematic evidence regarding the potential equity consequences of a proposed policy, before it is enacted.^7^ In theory, REIAs are intended to function as a policy tool for translating evidence on racial inequities into actionable guidance at a stage in the policy process when revisions remain possible. In practice, however, the role REIAs play in shaping legislative outcomes and the conditions under which they influence policy design remain poorly understood.

Existing research has primarily documented where REIAs have been adopted, the policy areas to which they apply, procedural features, and defining characteristics and quality of the assessments.^7^, ^8^, ^9^ However, less is known about how REIA ratings and conclusions are characterized and how they align with legislative outcomes. For example, questions include what conclusions REIAs generate, how those conclusions vary across policy domains, and whether the identification of potential equity harms inform modifications in proposed legislation. This gap is particularly relevant for informing emerging work in policy implementation science because it highlights how evidence on racial equity may be considered. REIAs are intended to influence policy decisions, but their effectiveness depends not only on analytic quality but also on institutional authority, political context, and accountability mechanisms that shape whether and how evidence is integrated into legislative decision‐making.^6^, ^8^, ^9^

The objective of this study was to characterize REIA ratings and legislative outcomes within a single US jurisdiction between 2021 and 2024. The distribution of projected racial equity impacts was characterized across policy domains, and bills identified as having adverse equity effects were assessed in terms of whether they were subsequently modified prior to enactment. By focusing on both REIA ratings and legislative responses, this analysis moves beyond questions of adoption to examine how equity‐focused policy tools function in real world governance. This study contributes to an emerging body of policy implementation science focused on how equity evidence is incorporated, or sidelined, within legislative processes. Understanding whether and how REIAs influence policy design is essential for strengthening their role as tools for informing the broader use of evidence‐based equity interventions in policymaking and ultimately advancing population health equity.

## Methods

### Data Source

REIAs in the District of Columbia (DC) were established under D.C. Law 23‐181, the Racial Equity Achieves Results (REACH) Act, to incorporate racial equity considerations into legislative and executive decision‐making processes.^10^ REIAs are conducted for all bills considered by the DC Council with the goal of identifying positive or adverse equity impacts prior to enactment and providing lawmakers with structured, evidence‐based insight to inform decisions.

The analysis in this study included all REIAs conducted for proposed legislation subject to DC's REIA mandate between January 2021 and December 2024, spanning two Council periods, namely, Period 24 (2021‐2022) and Period 25 (2023‐2024). Each Council period corresponds to a two‐year legislative session, during which bills are introduced, amended, and/or enacted. REIAs were drawn from a publicly available database generated through the racial equity mandate and represent the full universe of bills for which an assessment was required during the study period. REIAs are conducted by policy analysts using shared guidelines. Each REIA follows a structured format that includes a plain‐language overview of the proposed legislation; the background and historical context of the bill, including data and research relevant to the policy; an analysis of potential racial equity impacts; a “Further Considerations” section highlighting opportunities to improve equity outcomes; and an “Assessment Limitations” section that outlines analytic constraints and the nonbinding nature of the findings. These components collectively serve as the basis of and inform the determination of the bill's equity impact rating.

### Equity Impact Rating

Each REIA includes a rating of the bill's potential effects on racial equity, based on two dimensions of impact: symptoms of racial inequity (e.g., inequity in access to resources or services), and underlying structures that reinforce racial inequity (e.g., institutional rules or decision‐making processes).^11^ Ratings may address one dimension or both, depending on the bill's content. For this analysis, REIA ratings were grouped into four harmonized categories: positive, adverse, neutral, and inconclusive/negligible (Appendix 1). Positive ratings indicate potential or likely improvement or progress (e.g., “will improve,” “will likely improve,” “will likely make progress,” “will make progress,” or “potential to advance”). Adverse ratings indicate potential or likely harm or exacerbation (e.g., “will harm” or “will likely harm,” “will likely exacerbate,” “will exacerbate,” or “potential to exacerbate”). Neutral ratings indicate maintenance of the status quo (“maintain status quo”). Inconclusive or negligible ratings indicate minor effects or insufficient information to determine impact (“negligible” or “inconclusive impact”). Although original REIAs ratings distinguished between negligible impacts and inconclusive findings, these categories were combined for this analysis because both indicate an absence of clear directional evidence that a proposed policy would meaningfully improve or worsen racial inequities. REIAs from the 2 years in each Council Period were pooled. Although the scoring procedures were slightly modified, during this period, the rating categories were harmonized to ensure comparability (Appendix 1).

REIAs can be conducted at multiple points during the legislative bill process (e.g., initial introduction, committee consideration, markup). When multiple REIAs were conducted for a single bill across different stages of the legislative process, only the final REIA was retained to reflect the most updated evaluation of the legislation and to avoid duplication. Within a given REIA, multiple equity impact ratings may be assigned to different provisions of the bill. These ratings were treated as distinct observations in this analysis. A total of 324 REIAs were accessed from the publicly available database, of which 296 nonduplicative REIAs were included in the analysis. Several of these REIAs included multiple bill‐level equity ratings, resulting in 409 observations, which serve as the unit of analysis in this study (see Appendix 2).

### Approach

Each REIA was systematically reviewed and coded for policy domain, REIA equity impact rating, and legislative outcome. REIA ratings were categorized into one of eight policy domains reflecting their substantive focus: health and human services, criminal justice and public safety, education, economic development and labor, budget and fiscal policy, transportation and infrastructure, environmental policy, and technology and data governance (see Appendix 3 for definitions). For analytic purposes, each bill‐level equity impact rating was assigned a primary policy domain based on the central focus of the legislation or provision. Although some bills span multiple domains, assigning a single primary domain enabled consistent categorization across analyses. The distribution of equity impact ratings was examined across policy domains, and bills identified as having adverse equity impacts were assessed in terms of whether they were subsequently modified prior to enactment. Descriptive statistics (counts and percentages) were used to summarize REIA ratings overall and across policy domains and legislative outcomes. To ensure the reliability of the policy domain categorization, a subset of REIAs was independently coded by two members of the research team, with discrepancies resolved through consensus discussion. Legislative outcomes were characterized as follows: the bill became stalled or was not enacted, the bill was enacted without modification, or the bill was modified before enactment. The descriptive analyses focused on characterizing patterns in REIA ratings and legislative outcomes and did not estimate the causal relationships between REIA ratings and policy change.

## Results

### Equity Impacts Identified by Racial Equity Impact Assessments

**Figure 1:**
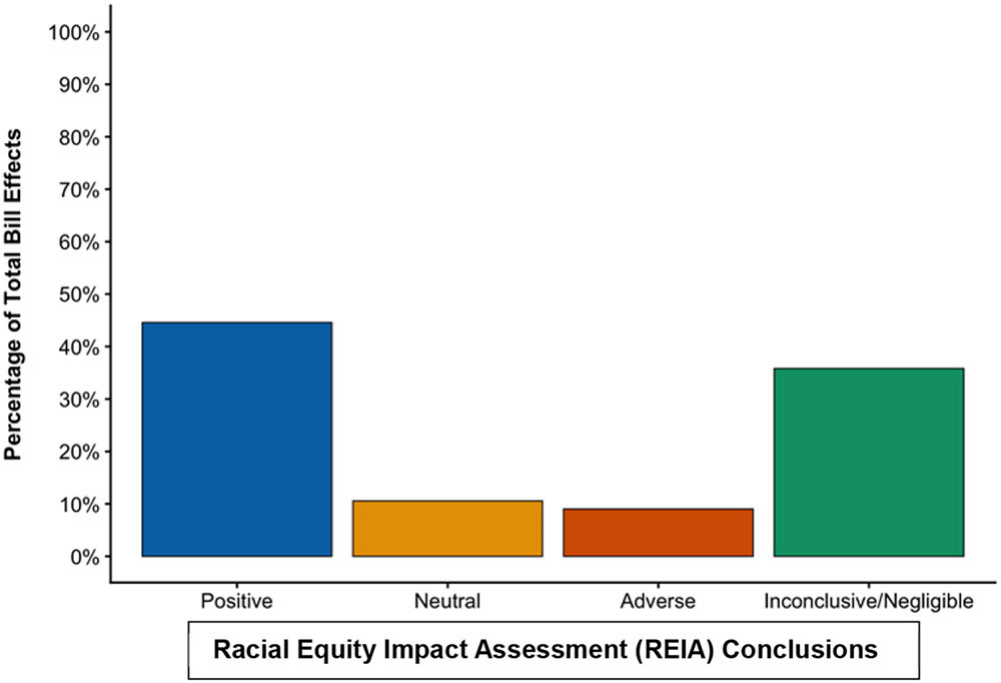
Distribution of Racial Equity Impact Conclusions (2021‐2024) [Colour figure can be viewed at wileyonlinelibrary.com]

Across the 409 bill‐level equity impact ratings derived from 296 final REIAs issued between 2021 and 2024, nearly half (46.4%) concluded that the proposed legislation was likely to advance racial equity (Figure 1). In contrast, 9.0% of assessments identified potential adverse effects that were expected to exacerbate racial inequities. More than one‐third of bill‐level ratings (34%) were categorized as inconclusive or negligible, indicating limited or indeterminate equity implications, and an additional 10% were classified as neutral.

**Figure 2:**
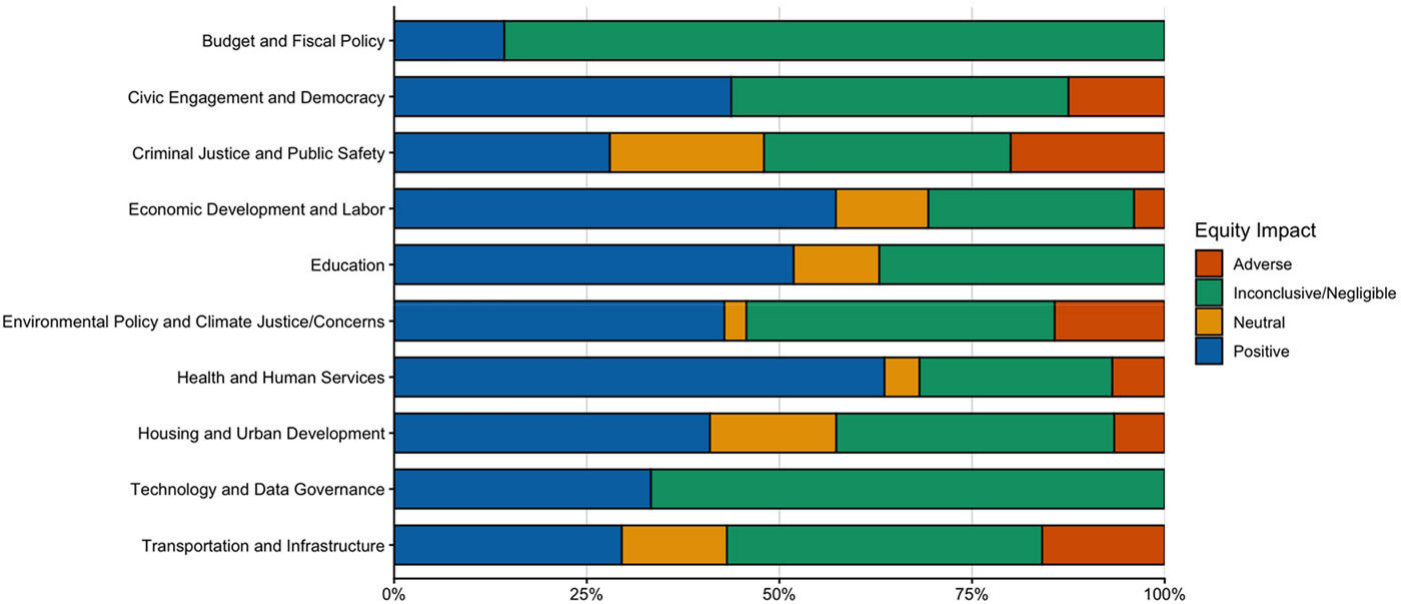
Racial Equity Impact Assessment Conclusions by Policy Domain (2021‐2024) [Colour figure can be viewed at wileyonlinelibrary.com] The unit of analysis is the REIA conclusion, not the total number of REIAs. A single bill may receive multiple REIA conclusions when different provisions of the legislation are assessed separately. Equity‐impact categories were defined by the issuing agency and were not classified by the authors. Inconclusive and negligible findings were combined because both indicate no clear directional equity impact. Percentages were calculated separately for each policy domain based on the total number of REIA conclusions in that respective domain.

The distribution of REIA conclusions varied substantially across policy domains (Figure 2). Health and human services policies were most frequently assessed as having positive racial equity impacts, with 63.6% of bill‐level effects projected to improve equity. Education and economic development and labor policies also exhibited relatively high proportions of positive findings. In contrast, budget and fiscal policy bills were least likely to receive positive equity assessments. Adverse equity findings were concentrated in a limited number of policy domains. Criminal justice and public safety legislation accounted for the largest share of adverse REIA ratings, followed by environmental policy and transportation and infrastructure. Patterns of inconclusive or negligible findings were also domain‐specific. Budget and fiscal policy, technology and data governance, and transportation and infrastructure had the highest proportions of assessments lacking clear directional equity effects. These findings suggest analytic and interpretive challenges when applying REIAs to highly technical, administrative, or indirect policy interventions, where pathways to outcomes may be less explicit or more difficult to assess.

### Legislative Responses to Adverse Equity Findings

**Figure 3:**
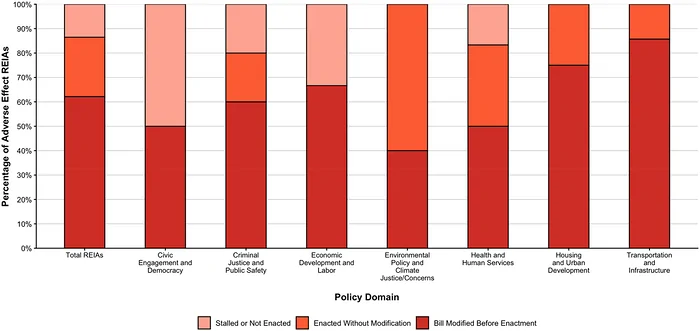
Policy Outcomes Following Adverse Racial Equity Impact Assessments, by Policy Domain (2021‐2024) [Colour figure can be viewed at wileyonlinelibrary.com] The unit of analysis is the REIA conclusion, not the total number of REIAs. A single bill may receive multiple REIA conclusions when different provisions of the legislation are assessed separately. It is not possible to determine whether modifications made before enactment were a direct result of the REIA recommendations or were due to other factors in the legislative process.

Legislative outcomes for bills receiving adverse REIA ratings are summarized in Figure 3. Across all policy domains, 62.2% of bills identified as likely to worsen racial equity were enacted with modifications prior to passage. Approximately one‐quarter of adverse bills were enacted without amendment, whereas 13.5% stalled, failed, or were not advanced to enactment. These descriptive patterns do not establish whether the REIA ratings directly influenced the legislative outcomes.

## Discussion

This study, to the authors’ knowledge, is among the first to provide a systematic empirical characterization of REIAs as a policy decision‐support tool within a single US jurisdiction. The analysis of 296 REIAs covering 409 bill‐level equity effects over a four‐year period reported herein demonstrates substantial variation in both the equity impacts identified by REIAs and the extent to which adverse findings were followed by legislative modifications. These findings offer insight into how equity‐focused assessments function within legislative processes and offer a foundation for understanding how their use and potential impact may be strengthened.

The REIA ratings in this study varied systematically across policy domain, reflecting differences in policy mechanisms. Positive equity findings were most concentrated in health and human services and in economic development and labor policy, where resource allocation and access‐enhancing provisions, workforce standards and investments, and service expansion create relatively direct links between policy design and equity outcomes. For example, the Certified Nurse Aide (CNA) Amendment Act of 2024 was expected to increase access to CNA certification and raise wages in a workforce disproportionately composed of Black and Latine adults. Similarly, the Medically Necessary Foods Coverage Act of 2022 requires health insurance plans to cover medically necessary foods for specific chronic health conditions, thereby reducing financial barriers. In these domains, REIAs frequently identified benefits tied to access, affordability, and labor protections, illustrating how equity assessments can surface population‐health–relevant impacts of upstream social policy.

A substantial proportion of REIAs concluded that proposed legislation would have an inconclusive or negligible equity impact, particularly in budget and fiscal policy and in technology and data governance. These findings underscore analytic and interpretive challenges inherent in applying equity assessments to policies that are more administrative, procedural, or technical in nature. The direct racialized effects of policies in these domains may be diffuse, less apparent, or difficult to measure, even though indirect or downstream impacts on equity may exist. It is possible that, in some cases, inconclusive or negligible findings may not represent analytic shortcomings of the REIA process; rather, they may genuinely reflect instances where the anticipated impact on racial equity is neutral. Yet, a growing body of scholarship challenges the assumption of neutrality in these policy domains, arguing that budgetary, regulatory, and procedural decisions are embedded within broader social and political contexts.^12^ These contexts can reproduce and perpetuate structural inequities even in the absence of explicitly racialized intent. Although refining REIAs in these domains may improve the identification of equity‐relevant impacts that are not immediately observable, important challenges remain in capturing diffuse and indirect pathways. Related work on equity impact assessments highlights the value of standardized reporting and communication tools to support the identification of equity‐relevant impacts.^13^

Adverse equity findings were disproportionately concentrated in criminal justice and public safety legislation, as well as in environmental and infrastructure policy. These domains are more likely to involve institutional discretion (e.g., changes to sentencing practices or modifications to carceral system operations), expanded enforcement authority, and heightened exposure to structural risk in ways that impact economic, health, social, and well‐being outcomes.^14^ Notably, REIAs identifying adverse impacts often included guidance aimed at mitigating harm. For example, in one REIA, the “Further Considerations” section emphasized how ambiguous statutory language can invite subjectivity and increase opportunities for biased decision‐making. Other REIAs highlighted the absence of race‐disaggregated data collection, underscoring the need for stronger data infrastructure to support equitable implementation and ongoing monitoring.

Across policy domains, a little over half of bills receiving adverse REIA ratings were enacted with modifications. Although it is not possible to determine whether these changes were made in direct response to REIA conclusions, the co‐occurrence of adverse assessments with both bill modification and the nonadvancement of subsequent revisions represents a descriptive pattern rather than evidence of a causal relationship. These findings highlight an important gap both in practice and in the literature, including a limited understanding of how equity‐focused policy tools are implemented and whether they achieve their intended effects.^15^ It is likely that a range of political, procedural, and contextual factors, independent of the REIAs, contributed to these legislative outcomes. However, it is possible that that these patterns suggest that REIAs may function as one input among the influences shaping legislative deliberation. The enactment of a substantial share of adverse bills without modification underscores the limits of authority of REIAs and the absence of formal accountability mechanisms requiring a response to equity findings. Importantly, these findings highlight the contingent role of REIAs within legislative decision‐making. They emphasize that the integration of equity evidence into policy change depends on institutional design, political context, and accountability structures and is not dependent solely on the production of analytic findings.

REIAs are conducted early in the legislative process, when revisions are still feasible, and they thus provide an opportunity for lawmakers to consider potential equity impacts. However, unlike fiscal impact statements, adverse REIAs are not binding and do not carry a formal requirement for legislative revision, which may limit their influence on policy outcomes and accountability with lawmakers. Without formal accountability mechanisms to ensure that adverse findings prompt action, jurisdictions risk advancing policies that perpetuate racial inequities in affected communities. These findings highlight a core challenge of equity governance related to the absence of formal institutional accountability mechanisms to ensure the uptake of such evidence within policy processes.

This study has several limitations. This descriptive analysis focused on a single jurisdiction with a well‐established REIA mandate and may not be generalizable to other settings with different political structures, contexts, priorities, or administrative capacity. As of March 2024, at least 11 states and several local government jurisdictions had adopted laws requiring REIAs.^16^ Variations in REIA scope, public availability, application across legislation, and standardization across jurisdictions limit cross‐jurisdictional comparative analysis. For example, in some jurisdictions, impact assessments apply only to criminal justice legislation, whereas other jurisdictions extend REIAs to budgeting and planning processes.^7^ In the analysis, a single primary policy domain was assigned to each bill‐level REIA rating, which may oversimplify complex legislation spanning multiple areas and obscure nuances across legislative provision. Although REIA ratings reflect a structured analytic process, some variation may occur across analysts. To our knowledge, formal inter‐rater reliability assessments are not conducted. However, policy analysts undergo standardized training and follow detailed guidelines to promote consistency in coding and rating practices. During the 2021‐2024 study period, minor modifications were made to the scoring rubric and documentation procedures that may limit comparability across years; however, the rating categories were harmonized to enhance consistency and minimize potential bias, which is likely modest. Lastly, a key limitation of this study relates to the inability to assess the specific nature of legislative revisions or determine whether bills that were stalled or never enacted were influenced by adverse REIA ratings. Anecdotally, conversations with legislative staff suggest that REIA findings may inform amendments or, in some cases, contribute to a bill not advancing, although these instances have not been systematically documented.

### Future Research

Future studies could build on this work in important ways. Quantitative research should examine whether REIA ratings are associated with measurable changes in social and population health outcomes. This could offer critical insight into the extent to which equity‐focused policy tools influence downstream inequities. Qualitative research, including interviews with REIA analysts, legislative staff, and policymakers, could help to clarify how equity findings are interpreted, negotiated, and acted upon during legislative deliberations, as well as to illuminate procedural barriers, political constraints, and opportunities to strengthen implementation. Future work should also examine the content of legislative modifications to assess whether and under what conditions REIA findings are integrated into the policy process. Expanding public access to REIAs and developing consistent reporting frameworks would further support cross‐jurisdictional comparisons and help identify conditions under which equity assessments most effectively shape policy. More broadly, this work highlights the need to better integrate equity‐focused policy tools and analyses within policy implementation science to illuminate how equity considerations are incorporated into policymaking and when such tools are most effective in shaping policy and practice.^15^ In this context, REIAs can be understood as equity‐informed implementation tools that anticipate and assess differential population impacts prior to policy enactment.^15^

Collectively, these findings suggest that REIAs offer a promising blueprint for embedding racial equity within local governance. By systematically documenting REIA ratings across policy domains and their alignment with legislative outcomes, this study provides critical evidence to help inform policymakers, advocates, and researchers seeking to advance population health equity. This research addresses a critical gap in understanding how equity‐focused policy procedures are operationalized in practice and how they shape policy processes in the context of structural racism and subsequent population health consequences.^5^ REIAs can serve as powerful tools for anticipating how proposed policies may improve, create, exacerbate, or reduce racial inequities. However, their effectiveness depends not only on analytic rigor, but also on institutional authority, political will, community engagement, and accountability structures that connect evidence to action. Maximizing the utility of REIAs will therefore require investment and commitment in governmental infrastructure to ensure that equity findings are meaningfully integrated into legislative deliberation and decision‐making processes.^8^, ^9^ Although this analysis focused on one jurisdiction, the patterns observed highlight considerations relevant to other contexts. Jurisdictions may benefit from applying REIAs more broadly across policy domains rather than limiting them to specific types of legislation. Strengthening accountability structures to ensure that equity assessments meaningfully inform decision‐making processes and making REIAs publicly available to enhance transparency, civic engagement, and shared learning may further support their impact. Amid growing political polarization and the retrenchment of equity‐focused policies and initiatives, the adoption, expansion, and refinement of REIAs across jurisdictions remains fragile. Ongoing quantitative and qualitative evaluation will be essential to strengthen their implementation, inform best practices, and generate new insights for the emerging field of racial equity policy implementation science.

## Funding/Support

None

## Conflict of Interest Disclosures

The authors have no conflicts of interest to disclose.

## Appendix 1

[Table milq70094-tbl-0001]

**Table A1 milq70094-tbl-0001:** Harmonization of REIA Ratings and Distributions by Council Period

Harmonized Categories	Original REIA Ratings	Years (Council Period)	
		2021‐2022 (24)	2023‐2024 (25)
Positive	Potential to advance	•	
	Will likely improve	•	
	Will make progress		•
	Will likely make progress		•
	Will improve	•	
Adverse	Potential to exacerbate	•	
	Will exacerbate	•	
	Will likely exacerbate	•	•
	Will likely harm	•	•
Neutral	Maintain status quo	•	•
Inconclusive/negligible	Inconclusive	•	•
	Negligible impact	•	•

## Appendix 2

[Table milq70094-tbl-0002]

**Table A2 milq70094-tbl-0002:** Distribution of REIAs and Bill‐Level Equity Impact Ratings by Year and Council Period

Council Period	Year	No. of REIAs	No. of Bill‐Level Ratings
24	2021	24	27
	2022	134	187
25	2023	53	73
	2024	85	122
Total		296	409

## Appendix 3

[Table milq70094-tbl-0003]

**Table A3 milq70094-tbl-0003:** Policy Domains and Definitions Used in REIA Coding

Policy Domain	Definition
Health and human services	Policies related to healthcare access, public health, behavioral health, and social service provision
Criminal justice and public safety	Policies related to policing, courts, corrections, and community safety systems
Education	Policies related to K–12 and higher education systems, including funding, access, and governance
Economic development and labor	Workforce policies, labor conditions, business regulations, and economic growth initiatives
Budget and fiscal policy	Government budgeting, taxation, and allocation of public resources
Transportation and infrastructure	Public transit, roadway systems, and built environment infrastructure
Environmental policy	Environmental protection, climate resilience, and sustainability initiatives
Technology and data governance	Data systems, digital governance, privacy, and algorithmic decision‐making
